# Functional Outcome After Lower Limb Amputation: Is Hyperhomocysteinemia a Predictive Factor? An Observational Study

**DOI:** 10.1097/MD.0000000000002167

**Published:** 2015-12-11

**Authors:** Stefano Brunelli, Augusto Fusco, Marco Iosa, Elena Ricciardi, Marco Traballesi

**Affiliations:** From the Santa Lucia Foundation, Scientific Institute for Research, Hospitalization and Health Care, Rome, Italy.

## Abstract

Lower limb amputation (LLA) is the drastic stage of peripheral arterial disease (PAD) where the hyperhomocysteinemia (H-HCY) seems to be a risk factor. Surprisingly, in literature the levels and the role of homocysteinemia (HCY) in persons with LLA are understudied. This study aims to investigate the level of HCY and its correlation with the functional outcomes after LLA.

A case–control study to analyze HCY levels in amputees admitted in a rehabilitation hospital during an investigation period of 1.5 years. Barthel Index was used to assess the functional outcome.

We enrolled 91 dysvascular amputees and 44 amputees for other reasons than PAD (controls). The mean level of HCY was found higher in dysvascular amputees (15.2 ± 7.5) compared to controls (11.0 ± 5.0, *P* < 0.0001) with a risk related ratio of 4.78. Normal Gaussian distribution of HCY was observed in controls, whereas in dysvascular amputees the data follow a double Gaussian distribution. Finally, a significant negative correlation was found between HCY and the effectiveness of rehabilitation (R = −0.37, *P* = 0.001) only in dysvascular amputees.

Dysvascular amputees had a level of HCY significantly higher than amputees without PAD. H-HCY seems to influence the functional outcomes of the rehabilitative treatment only in LLA due to PAD.

## INTRODUCTION

Due to the increase of life expectancy, adults are progressively more exposed to vascular diseases, like cerebrovascular, cardiovascular or peripheral diseases, both alone and concurrently.^1^ Peripheral arterial disease (PAD) is a chronic condition leading to arterial occlusion of the lower extremities often caused by atherosclerosis. The most severe form of PAD includes ulceration or gangrene, a preliminary step to lower limb amputation (LLA).^2^ One of the mainstays of PAD treatment is the managing of the risk factor. Among the modifiable risk factors, there are the increased levels of plasma homocysteine (HCY).^3–5^ Elevated plasma HCY is one of the suggested risk factors for endothelial dysfunction and vascular injury by increasing the oxidation of low density lipoprotein within vascular cells and tissues, preparing the atherosclerotic plaque formation.^6^

In literature, it has been strongly demonstrated an association between raised plasma HCY and an increased risk of developing cardiovascular diseases, including PAD.^7,8^ The metabolism of HCY depends on many factors including the availability of cofactors such as folate and vitamin B12 and the genes encoding enzymes involved in the HCY metabolism. A strong familial association in premature PAD has been found, suggesting an underlying genetic basis related to a modification of the activity of methylenetetrahydrofolate reductase (MTHFR), although other environmental factors, like smoking, may also be taken into account.^9,10^

Previous meta-analysis have suggested a causal relationship between hyperhomocysteinemia (H-HCY) and cerebro- and/or cardiovascular diseases.^11,12^ Pharmacological treatments with large doses of folic acid, pyridoxine hydrochloride (vitamin B6), and cyanocobalamin (vitamin B12) can lead to a decrease of the HCY levels in the blood, reducing mortality.^13^ However, it is debated whether the use of high doses of folic acid and B vitamins is more useful in terms of prevention or survival. Several interventional studies targeting to reduce HCY levels have not shown a consistent benefit on cardiovascular events or major events, as death, in a variety of high-risk patients,^14–17^ even if some benefits from vitamin intervention have been found for patients with stroke.^18^

Despite the large number of clinical observational and interventional trials on cardiovascular and cerebrovascular diseases, very few studies have been focused on implications in PAD, and particularly in the most compromised patients, such as the lower limb amputees. Previous reports have rated a prevalence of H-HCY between 27% and 32% in patients with chronic ischemia, with a slight male preponderance.^19^ An increased blood level of HCY is considered an independent risk factor for PAD and predictive of critical ischemia.^19^

No studies have certificated the HCY levels in persons after LLA and its possible impact on the rehabilitation outcomes. This study aims to investigate the levels of HCY in persons with dysvascular amputation at admission into a rehabilitation hospital and, as secondary outcome, to assess if these levels could be a prognostic factor of the functional outcome.

## MATERIALS AND METHODS

### Protocol and Participants

Each patient with unilateral dysvascular above or below knee amputation admitted to our inpatient rehabilitation unit was enrolled along a period of 1.6 years, independently by its time interval from the surgical event (PAD-Amp: persons affected by LLA due to peripheral arterial disease). As a control group, we enrolled patients admitted to the same rehabilitation unit after an LLA caused by a traumatic injury or neoplastic disease (NOPAD-Amp: persons with LLA due to diseases other than peripheral arterial disease). The sample size was chosen based on previous analogous study.^6^

The enrolled participants were analyzed for their HCY levels. For avoiding H-HCY due to other factors, we have excluded in both groups patients affected by alcohol abuse, comorbidities such as: hypothyroidism, psoriasis, systemic lupus erythematosus, rheumatoid arthritis, chronic renal failure, drug treatment with methotrexate, carbamazepine, phenytoin, isoniazid, and cardiopulmonary decompensation: these conditions themselves may determine H-HCY.^20^ We also excluded patients with a previous diagnosis of H-HCY treated with folate or B vitamins. We only enrolled patients whose clinical conditions did not contraindicate the rehabilitative training with the prosthesis.

The plasma concentrations of HCY were detected and defined according to the international standards, and as applied in previous analogous studies.^19,21^ The blood samples were taken at the bedside at 6:30 am in the morning by a registered nurse. A level of HCY more 13.0 μmol/L was defined as H-HCY. HCY levels were specified by making use of competitive immunoassay technique.

The functional status of inpatients amputees was assessed at admission and at discharge. The Barthel Index (BI) was used to assess the functional status. The BI is a 10-items scale that covers self-care for the evaluation of activities of daily living (ADL) (in particular, BI assesses the presence of fecal incontinence, presence of urinary incontinence, and the help needed with grooming, toilet use, bathing, feeding, and dressing) and mobility (help needed for ambulation, for transfers such as from chair to bed, and for stairs climbing). The score of each domain ranges from 0 to 10, therefore the total score ranges from 0 to 100: a score of 0 indicates total dependence in ADL and a score of 100, complete independence.^22^ The BI has been proven to have a valuable construct validity and to predict the long-term functional outcome. The duration of rehabilitation depended on the clinical conditions of the patient, and it was between 30 and 45 days. Individual physiotherapy exercises, including prosthetic training and occupational therapy, were performed for 60 minutes, twice a day, 5 days a week.

This protocol was approved by the local independent ethical committee and all enrolled patients gave written informed consent before the analysis.

### Data Analysis

Data have been summarized in terms of mean and standard deviation. Preliminary analysis between PAD-Amp and NOPAD-Amp has been performed using Mann–Whitney *U* tests. A forward binary logistic regression has been performed to highlight the dichotomized factors determining an H-HCY. The odds ratios reported below refer to the exponential value of β-coefficient of that regression, and we also reported the relevant 95% confidence interval (95% CI). The following factors have been evaluated (and dichotomized as follows): age (>65 years or not) sex (males vs. females), amputation level (above knee vs. below knee), time from amputation (>12 months or not), BI-score (>50 or not).

Test of Shapiro–Wilk was performed for testing the normality of data distribution. Data were finally grouped and Gaussian and double Gaussian fits were tested computing coefficient (R^2^) and adjusted coefficient of determination (R*adj*2). The correlation between the level of HCY and BI at admission and effectiveness of treatment in terms of BI-score were evaluated using the Spearman correlation coefficient (R). Effectiveness was computed as the percentage improvement between admission and discharge (score at discharge − score at admission) in respect of maximum achievable improvement (maximum scale score − score at admission). Critical alpha level was set at 0.05 for all the tests. SPSS 17.0 software (SPSS, Inc., Chicago, IL) was used for all statistical.

## RESULTS

Ninety-one PAD-Amp and 44 NOPAD-Amp (as control group, 27 amputees for traumatic injury, 16 for neoplastic disease) were enrolled in this study: demographical and clinical data are reported in Table 1. Some of these features resulted matched between the 2 groups at admission in our hospital (age, amputation level, BI-score), whereas some other did not (sex, time interval from surgery). Homocysteine level resulted significantly different between PAD-Amp and NOPAD-Amp.

**TABLE 1:**
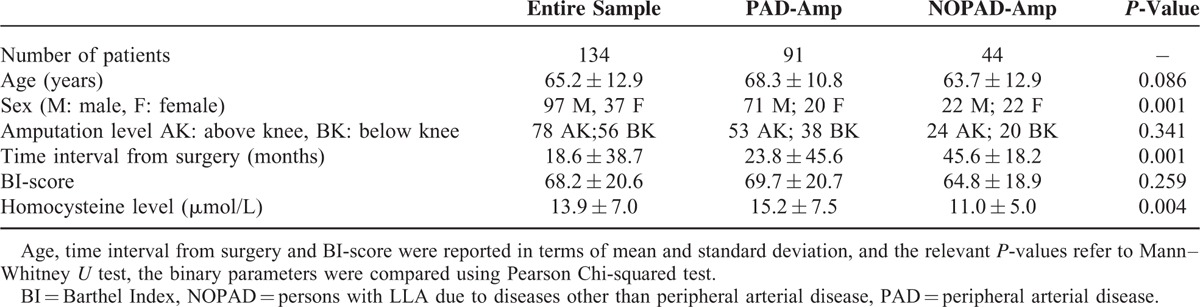
Demographic and Clinical Patients’ Characteristics at Admission in Our Hospital

A multivariate analysis (forward binary logistic regression) highlighted at the first step 3 factors potentially affecting the level of HCY: the group (PAD-Amp vs. NOPAD-Amp), age and sex of patients. The final model, that explained the 70% of the variance, identified that only group and age had a statistically significant role on HCY. All the other variables taken into account did not enter in the model (Table 2).

**TABLE 2:**
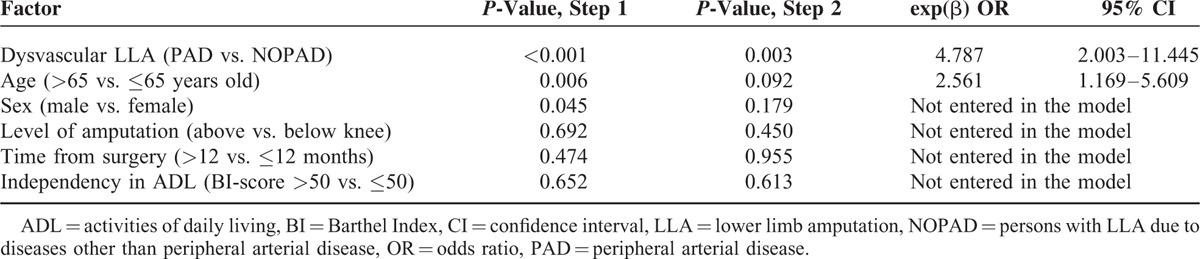
Results of Forward Binary Logistic Regression

The odds ratio related to the level of HCY between PAD-Amp and the control group was almost 5, whereas age was about 2.5. It means that there was a prevalence of H-HCY more than 5 times higher in PAD-Amp with respect to NOPAD-Amp where the 2 groups were matched for other factors. At the same time, elderly patients showed a prevalence of H-HCY more than twice higher than younger patients.

On the other hand, when HCY is considered as an exposition factor and PAD-Amp as cases (instead of the contrary as above), the prevalence of being amputated with H-HCY resulted with an odds ratio similar to the one reported above (OR = 4.58; 95% CI = 1.90–11.05).

The plasma level of HCY did not result normally distributed in PAD-Amp (*P* = 0.001, test of Shapiro–Wilk for normality), whereas it resulted normally distributed in NOPAD-Amp (*P* = 0.89).

Analogously, the coefficient of determination of a Gaussian fit, that resulted in, a normal distribution, was higher in NOPAD-Amp (R^2^ = 0.88), and lower in PAD-Amp (R^2^ = 0.70). In PAD-Amp, a double Gaussian distribution fitted the data (R^2^ = 0.90) better than simple Gaussian (R*adj*2 = 0.80 vs. 0.63).

**FIGURE 1:**
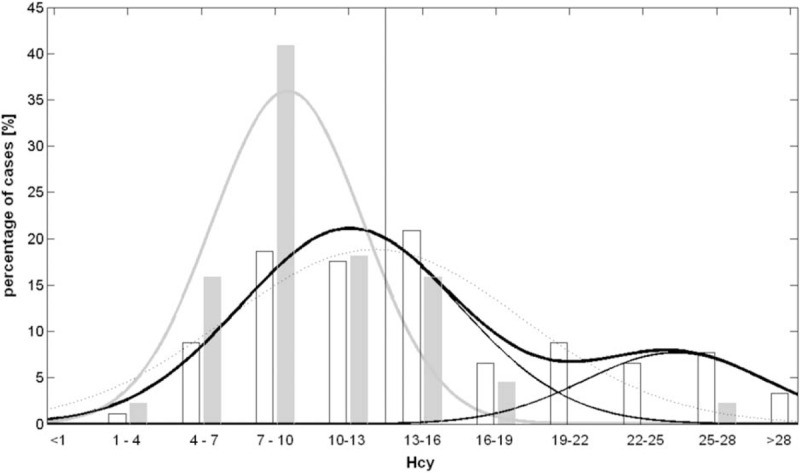
Histogram plot of HCY for PAD-Amp (black empty bars) and NOPAD-Amp (gray empty bars) in 11 ranges (the higher value for each range should be considered appertaining to the successive range). Gaussian fits are also reported: for NOPAD-Amp wide gray line and for PAD-Amp black lines (dot line: simple Gaussian, wide solid line: double Gaussian, thin solid lines: the 2 Gaussian distributions forming the double one).

As shown in Figure 1, the peak of HCY distribution in NOPAD-Amp was in the range of 7 to 10 μmol/L. The double Gaussian distribution of PAD-Amp had one peak in the range of 10 to 13 and the other one between 22 and 28 μmol/L. This bimodal distribution cannot be attributed to the sum of the effects of HCY and age in some subjects. In fact, the mean age of PAD-Amp in the range 10 to 13 (first peak) was 70.2 ± 8.9, that in the range 13 to 22 was 68.1 ± 12.5 (reduced prevalence), and that in the range 22 to 28 (second peak) was 69.4 ± 8.6 (no significant differences in these 3 ranges, *p *= 0.84).

**FIGURE 2:**
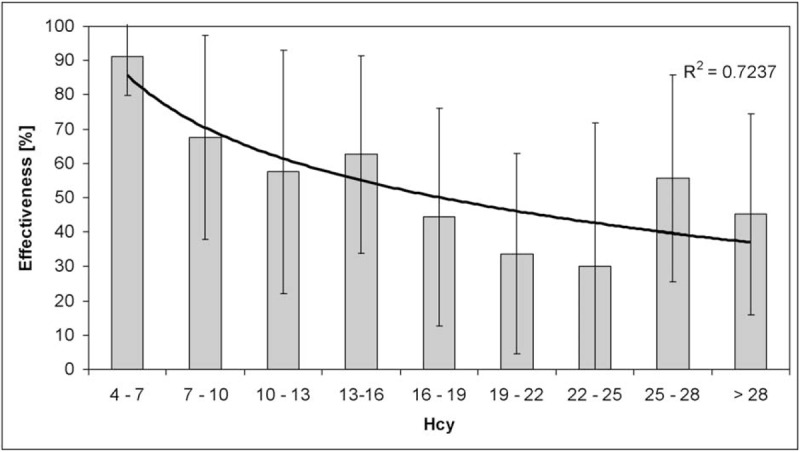
Histogram plot of BI effectiveness of intervention (mean ± standard deviation) related to HCY in PAD-Amp. A logarithmic fit is superimposed and its coefficient of determination was reported.

As a secondary aim, the levels of HCY did not significantly result correlated with the BI-score at admission for both groups (R = −0.01, *P* = 0.92 for PAD-Amp; R = 0.08, *P* = 0.58 for NOPAD-Amp). No significant correlation was found in NOPAD-Amp between HCY levels and BI-score at discharge (R = −0.11, *P* = 0.47) and BI effectiveness (R = −0.12, *P* = 0.41). Conversely, a significant correlation was found in PAD-Amp between HCY levels and the effectiveness of rehabilitation (R = −0.37, *P* = 0.001), that is a lower improvement in terms of BI-score for higher levels of HCY. This result was confirmed by a lower effectiveness in subjects with H-HCY (47.88 ± 32.61%) with respect to those with HCY less than 13 μmol/L (66.58 ± 31.95%, *P* = 0.02, *U* test). Figure 2 reports the mean of effectiveness for grouped levels of HCY: a logarithmic decreasing trend was observed for higher levels of HCY .

## DISCUSSION

Our study aimed to investigate the levels of HCY and the correlation of elevated levels of HCY with the functional rehabilitative outcome in a sample of PAD-Amp and NOPAD-Amp. Our results clearly showed a significantly higher prevalence of H-HCY in PAD-Amp than in NOPAD-Amp, confirmed by a multivariate analysis.

Even if PAD is considered as one of the major public health concerns worldwide, the role of HCY is surprisingly underestimated in the following and drastic stage, the LLA. This could be due to the fact that conflicting interpretations have arisen about the role of HCY and treatment of H-HCY. In fact, studies observing the effects of these treatments in long-term outcomes have been debated for their inherent limitations, both by a methodological and physiological point of view.^18^ It has been suggested that the plasmatic levels of HCY have a nonlinear correlation with the damage of the human vascular tree. The effect of the treatments for H-HCY cannot be detected at the vascular tree level because the modifications are too slight. Therefore, previous studies suggested that H-HCY may mark the existence of vascular disease rather than causes it.^16,18,23^ Our results cannot clarify if H-HCY is a cause or an effect; however, our results clearly showed that the risk to have H-HCY was almost 5 times significantly more frequent in PAD-Amp than in NOPAD-Amp.

This risk is considerably higher than the one for patients with lower limb ischemia, reported in literature with percentages among 27% and 32%.^6,19^ It has to be noted that persistent high levels of HCY in a period of 3 years lead to a major amputation risk, with an amputation in around half of the cases.^19^ The level of HCY in the blood was not reduced in subjects whether considered an old amputation or a recent one, as confirmed by our results. The other factor resulting statistically significant was age, with H-HCY more common in older amputees. Other factors, such as sex and or level of amputation, did not affect the level of HCY. Previous studies just reported a slight male preponderance.^6,19^ We also found a significant effect of sex at the first step of regression analysis, but it was not maintained in the final model.

The analysis of the distribution of HCY level revealed a bimodal distribution in the PAD-Amp. Because none of the above factors resulted affecting significantly this level with the exception of age, and the age was not different among the 2 peaks and the central dip of that distribution, some other reason should be found in further studies for this bimodal distribution. A possibility is that some PAD-Amp have a normal distribution of HCY similar to those of controls subjects and other PAD-Amp have a higher mean of HCY level (between 19 and 28) around which data were normally distributed. However, also the first Gaussian curve forming the bimodal distribution of PAD-Amp was shifted versus higher values of HCY (with a mean peak in the range 10–13) with respect to the NOPAD-Amp.

Another hypothesis is that HCY could be high in all PAD-Amp, subdividing subjects only in those having a low and a high alteration of its level. Globally, the probability to find an H-HCY in PAD-Amp was almost 5 times higher than in NOPAD-Amp. This finding means that the surgical event by itself did not influence on the HCY level but we can speculate that high HCY levels were related to PAD.

An important result of this study was related to the correlation of HCY levels with the functional outcome of the PAD-Amp. In fact, we have found that a condition of H-HCY can influence negatively the effectiveness of rehabilitation in PAD-Amp. The same analysis in the NOPAD-Amp group did not show this correlation. At the best of our knowledge, no previous studies have shown this relationship. In a recent interventional study, Waters et al^24^ have demonstrated that treating H-HCY with folic acid and vitamin B12 before revascularization procedures improve the clinical outcomes for patients with critical limb ischemia. We could hypothesize that the higher the level of HCY, the higher the general impairment, for example, heart and vascular disease, could reduce the capability to gain high performance in the ADL as shown by low BI effectiveness.

These data should be helpful for designing a further prospective trial for reducing the H-HCY in patients with LLA. The presence of LLA in vascular disease increases the morbidity and mortality of patients with PAD, up to 36% at 1 year and up to 48% at 5 years in these patients.^25^ At the moment the role of treatments based on high dose of multivitamins is discussed. Many international trials for major events prevention have failed in the primary (death from cardiovascular causes, myocardial infarction or stroke) and secondary outcomes (comorbidities).^15–18^ Also in a prospective trial in patients with kidney transplant, these treatments have failed in reducing both the following primary composite arteriosclerotic cardiovascular disease's outcome as myocardial infarction, stroke, cardiovascular disease death, including lower-extremity arterial disease and all-cause mortality too.^26^ At the same time, the considered factors influencing the postoperative functional status of patients with major LLA are related to demographic (age, gender, race) and clinical features (diabetes and/or peripheral vascular disease, presence of comorbidities) and the ambulatory and living status before the surgical procedure.^27–29^ The role of HCY before or after LLA in terms of functional status has never been taken into consideration. Future observational trials should also consider the levels of HCY to determine the possible role in the achieved outcomes after an LLA, addressing the following studies to interventional trials.

Due to inclusion criteria we excluded from the study 5 PAD-Amp with anamnestic H-HCY because they were already treated with folate and B vitamins. It is surprising that a population with severe PAD has not been sufficiently studied previously (only 7% of the whole sample of PAD-Amp) to exclude the H-HCY as a risk factor. This result suggests that HCY blood level was poorly taken into account in people with PAD.

## LIMITATIONS

The main limit of this study is the heterogeneity of the samples that include more levels of LLA (above or below knee) that certainly influenced the functional outcome. Another limit is the global evaluation of the functional status (by means of BI) and not a detailed evaluation of prosthetic use (eg, by Locomotor Capability Index). However, in literature, other authors used only the BI as an index of outcome in a mixed population of transfemoral and transtibial amputees.^30^

Finally this study lacks of a reevaluation of HCY at the end of the rehabilitative treatment. It should be noted that enrolled persons with high levels of HCY at admission were treated with appropriate doses of folate and B vitamins.

## CONCLUSIONS

In conclusion, our results showed as PAD-Amp have a level of HCY significantly higher than NOPAD-Amp. H-HCY seems to influence the outcomes of rehabilitation only in dysvascular amputees. Further studies should take into account the level of HCY both as a possible marker in dysvascular amputees and as a possible risk factor of amputation. HCY levels should be monitored and put in relationship with functional outcomes in studies with long follow-up. Moreover, further studies should evaluate if H-HCY is a further predictive factor in rehabilitation of amputees for vascular diseases.^31^
